# Preservation of forensic traces by Nursing in emergency services: a scoping review

**DOI:** 10.1590/1518-8345.5849.3540

**Published:** 2022-07-08

**Authors:** Rute Xavier Silva, Carlos Adriano Alves Ferreira, Guilherme Guarino de Moura Sá, Rafaella Queiroga Souto, Lívia Moreira Barros, Nelson Miguel Galindo-Neto

**Affiliations:** 1 Instituto Federal de Educação, Ciência e Tecnologia de Pernambuco, Campus Pesqueira, Pesqueira, PE, Brasil.; 2 Instituto Federal de Educação, Ciência e Tecnologia de Pernambuco, Campus Belo Jardim, Belo Jardim, PE, Brasil.; 3 Universidade Federal da Paraíba, João Pessoa, PB, Brasil.; 4 Universidade da Integração Internacional da Lusofonia Afro-Brasileira, Departamento de Enfermagem, Redenção, CE, Brasil.

**Keywords:** Nursing, Forensic Nursing, Expert Testimony, Emergency Nursing, Emergencies, Review, Enfermagem, Enfermagem Forense, Prova Pericial, Enfermagem em Emergência, Emergências, Revisão, Enfermería, Enfermería Forense, Testimonio de Experto, Enfermería de Urgencia, Urgencias Médicas, Revisión

## Abstract

**Objective:**

to map the scientific production on the preservation of forensic traces by Nursing professionals working in emergency services.

**Method:**

a scoping review, with searches for studies carried out in six databases, in the gray literature available in *Google Scholar* and in the references of the studies selected. For analysis, the data reduction method was adopted.

**Results:**

26 studies were included, organized into five categories: 1) Nursing professionals’ knowledge on the preservation of forensic traces; 2) Procedures performed by Nursing to preserve traces in the victim’s body; 3) Procedures performed by Nursing to preserve traces in the victim’s belongings/objects; 4) Procedures performed by Nursing to document traces; and 5) Actions to maintain the chain of custody performed by Nursing.

**Conclusion:**

the studies showed situations in which the emergency nurse may act in the preservation of forensic traces present in the victim’s body and in objects, as well as in the registration of traces, verifying the role of Nursing to ensure integrity of the chain of custody, especially in situations of aggression, firearm injury, sexual violence, child abuse and assistance to trauma victims.

## Resumo

**Objetivo:**

mapear a produção científica sobre a preservação de vestígios forenses pelos profissionais de enfermagem que atuam nos serviços de emergência.

**Método:**

revisão de escopo, com buscas dos estudos realizadas em seis bases de dados, na literatura cinzenta disponível no *Google Scholar* e nas referências dos estudos selecionados. Para análise, adotou-se o método de redução de dados.

**Resultados:**

foram incluídos 26 estudos que foram organizados em cinco categorias: 1) Conhecimento dos profissionais de enfermagem sobre a preservação de vestígios forenses; 2) Procedimentos realizados pela enfermagem para preservação de vestígios no corpo da vítima; 3) Procedimentos realizados pela enfermagem para preservação de vestígios em pertences/objetos da vítima; 4) Procedimentos realizados pela enfermagem para documentação dos vestígios; e 5) Ações de manutenção da cadeia de custódia realizada pela enfermagem.

**Conclusão:**

os estudos mostraram situações em que o enfermeiro de emergência pode atuar na preservação de vestígios forenses presentes no corpo da vítima e em objetos, bem como no registro dos vestígios, verificando-se a atuação da enfermagem para garantir a integridade da cadeia de custódia, principalmente em situações de agressão, de ferimento com arma de fogo, violência sexual, abuso infantil e na assistência a vítimas de trauma.

## Resumen

**Objetivo:**

mapear la producción científica sobre la preservación de rastros forenses por profesionales de enfermería que trabajan en los servicios de emergencias.

**Método:**

revisión de alcance, con búsqueda de los estudios realizados en seis bases de datos, en la literatura gris disponible en *Google Scholar* y en las referencias de los estudios seleccionados. Para el análisis, se adoptó el método de reducción de datos.

**Resultados:**

se incluyeron 26 estudios organizados en cinco categorías: 1) Conocimiento de los profesionales de enfermería sobre la preservación de rastros forenses; 2) Procedimientos que realizan los enfermeros para preservar rastros en el cuerpo de la víctima; 3) Procedimientos que realizan los enfermeros para preservar rastros en las pertenencias/objetos de la víctima; 4) Procedimientos que realiza el personal de enfermería para documentar los rastros; y 5) Acciones que llevan a cabo los enfermeros para mantener la cadena de custodia.

**Conclusión:**

los estudios demostraron que hay situaciones en las cuales el enfermero de emergencias puede actuar para preservar los rastros forenses presentes en el cuerpo de la víctima y en los objetos, así como también registrar los rastros, lo que comprueba la actuación del enfermero para garantizar la integridad de la cadena de custodia, particularmente en situaciones de agresión, heridas con arma de fuego, violencia sexual, abuso infantil y asistencia a víctimas de trauma.

**Highlights**

(1) The Nursing workforce in emergency services contributes to preserve forensic traces.

(2) There is a gap in Brazilian evidence on the preservation of traces in emergency services.

(3) Traces on the victim’s body and objects can be preserved by Nursing.

(4) Forensic traces found by Nursing in the emergency services must be documented.

## Introduction

Emergency health services often act in the care of victims of crime situations; thus, they have in these environments a privileged opportunity to identify, collect and preserve forensic traces^1^ ^-^ ^2^. These traces can include palm and plantar fingerprints; biological elements such as blood, semen, saliva, hair, bones, teeth, hair and vaginal secretions; and physicochemical traces such as chemicals, projectiles, melee weapons, firearms and sharp objects or instruments^3^ ^-^ ^6^.

Nursing professionals, who are on the front line in the care of patients in emergency services, in addition to having specific attributions to preserve life and reduce sequelae, must collaborate with the preservation of the traces present in the victim, in the possible aggressor, in the objects and at the crime scene^3^. Such traces, of high presence in Nursing care in emergency services, are essential elements for the success of the criminal investigation and for integrity of the chain of custody, as such chain consists of maintenance and documentation of traces, from their identification, collection, possession and handling until their disposal^1^.

Collaboration of the Nursing professionals in forensic investigation can prevent the unnecessary loss or destruction of evidence; however, lack of knowledge in these professionals who work in the emergency services about proper preservation of traces exerts an impact on the work of the expert team^7^.

Although the action towards victims of crimes occurs in the Nursing practice, most of the professionals do not have access to information on the theme^3^. Lack of training or moments of permanent education on the preservation of forensic traces, a content transversal to the Forensic Nursing specialty, results in the non-association of forensic care as inherent to the Nursing actions in emergency services^2^.

In this context, knowing the scientific production about the preservation of forensic traces by Nursing professionals who work in emergency services is relevant, as it may enable nurses to access scientific information about the preservation of traces, given the growing reality of situations involving crime in emergency services. Thus, it is pointed out that this study will allow for the compilation and construction of new knowledge, which can be used in the training and qualification of Nursing professionals who work in the emergency services, in order to empower them with regard to the correct performance in situations in which forensic remains need to be preserved. In addition, although the review focus is on the Nursing team, it is noteworthy that this study is of potential interest to the multidisciplinary health team and managers whose professional performance permeates the context of emergency services.

Thus, this study mapped the scientific production on preservation of forensic traces by Nursing professionals working in emergency services.

## Method

### Type of study

This is a scoping review that followed the stages recommended by the Joanna Briggs Institute (JBI)^8^ and the Preferred Reporting Items for Systematic reviews and Meta-Analyses extension for Scoping Reviews (PRISMA-ScR) checklist^9^. The review was developed in five stages: identification of the research question; survey of relevant studies; selection of the studies; data mapping; and presentation of the results^10^.

### Study setting

This review was conducted in six databases: National Center for Biotechnology Information (NCBI/PubMed); Excerpta Medica Database (EMBASE); Cumulative Index to Nursing and Allied Health Literature (CINAHL); Web of Science; *Literatura Latino-Americana e do Caribe em Ciências da Saúde* (LILACS) via *Biblioteca Virtual em Saúde*(BVS); and *Índice Bibliográfico Español en Ciencias de la Salud* (IBECS). 

### Period

The study was conducted between August and October 2021.

### Population

The study population consisted of the 190 scientific articles found in the searches in the databases and in the gray literature available on *Google Scholar*.

### Selection criteria

Articles with different types of research addressing the preservation of forensic traces by Nursing professionals in emergency services were included, without limitation regarding language or year of publication. For exclusion of the studies, we adopted the criteria of letters to the editor, abstracts of annals of events and not presenting information that contemplated the population, concept and context of interest of this study.

### Study variables 

The study variables were as follows: title of the article; year of publication; country; journal; language; objective; type of study; public studied; type of injury; location of the trace (crime scene, objects and belongings, victim’s body or others); and information on the preservation of forensic traces. After data extraction, the findings of both reviewers were compared, any and all discrepancies were resolved and the information was grouped into a single table.

### Instruments used to collect the information

The diverse information extracted from the studies was recorded in a data collection instrument adapted from a form recommended by the JBI, organized in a Microsoft Excel 2001 spreadsheet^11^.

### Data collection

In a previous search in the JBI database, no reviews were found to investigate the issue. 

For elaboration of the research question, the PCC (Population, Concept and Context)^8^ mnemonic was used, where: P - Nursing professionals, C - Preservation of forensic traces and C - Emergency service. Thus, the research question adopted was as follows: “What scientific evidence is available on the preservation of forensic traces by Nursing professionals working in emergency services?”

The searches took place in August 2021, through remote access to the databases, from registration in the journals portal of the Coordination for the Improvement of Higher Education Personnel (*Coordenação de Aperfeiçoamento de Pessoal de Nível Superior*, CAPES), via the Federated Academic Community (*Comunidade Acadêmica Federada,* CAFe), at the login of the Federal Institute of Education, Science and Technology of Pernambuco (*Instituto Federal de Educação, Ciência e Tecnologia de Pernambuco*, IFPE).

From the research question, the descriptors of the Medical Subject Headings (MeSH), EMBASE Subject Headings (EMTREE), CINAHL Headings and Descriptors in Health Sciences (*Descritores em Ciências da Saúde*, DeCS) were selected. In addition to that, uncontrolled descriptors were used to broaden specificity of the search.

Figure 1 presents the descriptors used in each database, as well as the use of the Boolean operators in the search for high sensitivity.

**Figure 1:** Expressions of the searches in the databases. Pesqueira, PE, Brazil, 2021

Database	Search terms
NCBI/PubMed^*^	(“Forensic Nursing”[MeSH Terms] OR “Emergency Nursing”[MeSH Terms] OR “Nursing”[MeSH Terms] OR “Nurses”[MeSH Terms]) AND (“Forensic Sciences”[MeSH Terms] OR “preservation, biological”[MeSH Terms] OR “Preservation of forensic traces”[All Fields]) AND (“Emergencies”[MeSH Terms] OR “Emergency Treatment”[MeSH Terms] OR “Emergency Medical Services”[MeSH Terms] OR “Emergency Relief”[All Fields])
EMBASE^†^	‘forensic nursing’/exp OR ‘emergency nursing’/exp OR ‘nursing’/exp OR ‘nurses’/exp AND ‘forensic sciences’/exp OR ‘preservation, biological’/exp OR ‘preservation of forensic traces’ AND ‘emergencies’/exp OR ‘emergency treatment’/exp OR ‘emergency medical services’/exp OR ‘emergency relief’
CINAHL^‡^	MH “Forensic Nursing” OR MH “Emergency Nursing” OR TX “Nursing” OR MH “Nurses” AND MH “Forensic Sciences” OR MH “preservation, biological” OR TX “Preservation of forensic traces” AND MH “Emergencies” OR MH “Emergency Treatment” OR MH “Emergency Medical Services” OR TX “Emergency Relief”
Web of Science	TOPIC (“Forensic Nursing”) OR TOPIC: (“Emergency Treatment”) OR TOPIC: (Nursing) OR TOPIC: (Nurses) AND TÓPIC: (“Forensic Sciences”) OR TOPIC: (“preservation, biological”) OR TOPIC: (“Preservation of forensic traces”) AND TOPIC: (Emergencies) OR TOPIC: (“Emergency Treatment”) OR TOPIC: (“Emergency Medical Services”) OR TOPIC: (“Emergency Relief”)
LILACS^§^ IBECS^‖^	((mh:(“Forensic Nursing”)) OR (“Enfermagem Forense”) OR (“Enfermería Forense”) OR (mh:(“Emergency Nursing”)) OR (“Enfermagem em Emergência”) OR (“Enfermería de Urgencia”) OR (mh:(Nursing)) OR (Enfermagem) OR (Enfermería) OR (mh:(Nurses)) OR (“Enfermeiras e Enfermeiros”) OR (“Enfermeras y Enfermeros”)) AND ((mh:(“Forensic Sciences”)) OR (“Ciências Forenses”) OR (“Ciencias Forenses”) OR (mh:(“preservation, biological”)) OR (“Preservação Biológica”) OR (“Preservación Biológica”) OR (“Preservation of forensic traces”) OR (“Conservación de rastros forenses”) OR (“Conservación de rastros forenses”)) AND ((mh:(Emergencies)) OR (Emergências) OR (“Urgencias Médicas”) OR (mh:(“Emergency Treatment”)) OR (“Tratamento de Emergência”) OR (“Tratamiento de Urgencia”) OR (mh:(“Emergency Medical Services”)) OR (“Serviços Médicos de Emergência”) OR (“Servicios Médicos de Urgencia”) OR (“Emergency Relief”) OR (“Socorro de Urgência”) OR (“Socorro de Urgencia”))

^*^NCBI/PubMed *=* National Center for Biotechnology Information; ^†^EMBASE *=* Excerpta Medica Database; ^‡^CINAHL *=* Cumulative Index to Nursing and Allied Health Literature; ^§^LILACS = *Literatura Latino-Americana e do Caribe em Ciências da Saúde*; ^||^IBECS = *Índice Bibliográfico Español en Ciencias de la Salud*

The results obtained in the databases were exported to the Rayyan reference manager, developed by the Qatar Computing Research Institute (QCRI)^12^, for removal of duplicates, selection and screening of THE studies by two researchers, independently and masked, and the divergences were resolved with the participation of a third examiner. After the search developed according to the strategy outlined above, the studies were selected. In addition, there was a search in the gray literature available on *Google Scholar* and a survey of potentially eligible articles was made in the reference lists of the studies selected. Reading of titles and abstracts was performed. The studies that met the inclusion criteria were analyzed by reading the manuscripts in full. Finally, manual searches were performed in the references of the studies included.

### Data treatment and analysis

For analysis, the data reduction method was adopted, which aims at conceptually classifying the results after critical reading^13^. For the review report, the Preferred Reporting Items for Systematic reviews and Meta-Analyses extension for Scoping Reviews (PRISMA-ScR) were followed^9^.

### Ethical aspects

As the studies used were of public domain access, there was no need to submit the study to the Research Ethics Committee.

## Results

**Figure 2:**
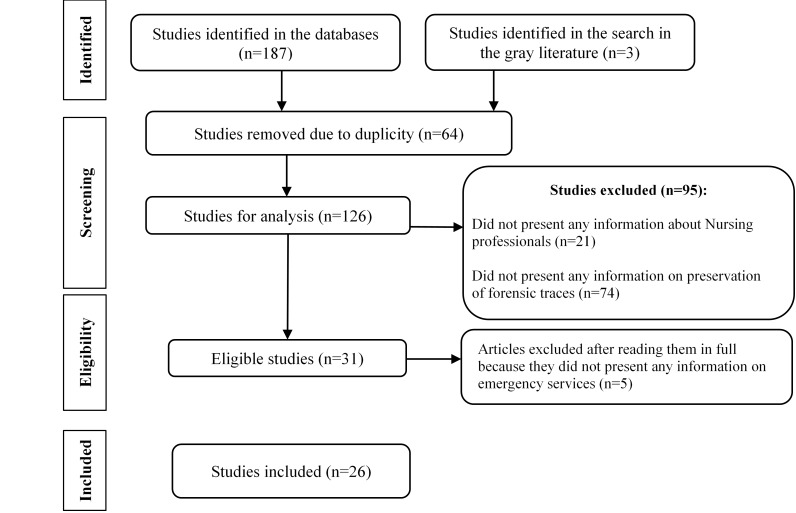
Flowchart of the article selection process for the scoping review. Pesqueira, PE, Brazil, 2021

A total of 190 articles were identified, of which 111 were found in EMBASE, 73 in PubMed, three in Web of Science and three by consulting *Google Scholar*. In the CINAHL, LILACS, BVS and IBECS databases, no studies were identified in the search to constitute the sample. After exclusion of the duplicate studies, 126 articles were evaluated for eligibility by the researchers, remaining 26 articles as shown in the flowchart in Figure 2.

The 26 articles analyzed were published in English. Regarding the countries where the studies were developed, there was predominance of the United States with 16 (61.5%), three from Canada (11.5%), two from Brazil (7.7%), two from Turkey (7.7%), one from Australia (3.8%) and one from Sweden (3.8%). Of the studies selected, 12 (46.2%) were cross-sectional; three (11.54%) were narrative reviews; two (7.6%) consisted of literature reviews; two (7.6%) were retrospective and longitudinal studies; one (3.8%) was an experience report; one (3.8%) was a case study; one (3.8%) was a prospective observational cut; one (3.8%) was a descriptive study (pilot); one (3.8%) was a retrospective study; one (3.8%) was a reflective study; and one (3.8%) was a brief communication. The characteristics of the studies are presented in Figure 3.

**Figure 3:** Characteristics of the studies that comprised the scoping review sample, according to title of the article, journal/country, study design, participants and/samples. Pesqueira, PE, Brazil, 2021

Country/Year of publication	Journal	Study design	Participants
United States/1991^14^	Critical Journal Care Nursing	Narrative review	Forensic nurses
United States/1996^15^	Tennessee Nurses Association	Cross-sectional	Emergency health professionals
United States/1998^16^	Journal of Emergency Nursing	Experience report	Sexual assault response team
Canada/1999^17^	Accident and Emergency Nursing	Case study	Emergency nurse and a police officer
Australia/2004^18^	Accident and Emergency Nursing	Literature review	Emergency nurses
Canada/2005^19^	Journal of Forensic Nursing	Cross-sectional	Sexual assault nurse examiner
United States/2005^20^	Trauma, Violence & Abuse	Literature Review	Sexual assault nurse examiners
United States/2007^21^	The Journal of Emergency Medicine	Prospective observational cohort	Emergency nurses
United States/2008^22^	Journal of Forensic Nursing	Descriptive (pilot)	Emergency and intensive care unit nurses
South Africa/2009^23^	Journal of Emergency Nursing	Cross-sectional	Emergency nurses from South Africa
Canada/2009^24^	Journal Of Emergency Nursing	Retrospective	Emergency nurses
United States/2010^25^	Critical Care Nursing	Narrative review	Emergency trauma nurses and operating rooms
United States/2010^26^	Critical Care Nursing	Cross-sectional	Emergency nurses
United States/2012^27^	Journal of Forensic Nursing	Cross-sectional	Physicians and nurses in the Emergency sector
United States/2012^28^	Journal of Forensic Nursing	Longitudinal and retrospective	Pediatric sexual assault nurse examiners
United States/2014^29^	Journal of Emergency Nursing	Reflective	Emergency nurses
Sweden/2014^30^	Journal of Clinical Nursing	Cross-sectional	Emergency nurses
United States/2015^31^	Critical Care Nursing	Longitudinal and retrospective	Emergency and Intensive Care Unit nurses
Turkey/2015^6^	Turkish Journal of Emergency Medicine	Cross-sectional	Nurses, Ambulance Technician and Emergency Physicians
United States/2015^32^	Critical Care Nursing	Cross-sectional	Emergency nurses
United States/2016^33^	Journal of Forensic Nursing	Narrative review	Emergency nurse and health professionals
United States/2018^34^	Emergency Nurses Association	Brief communication	Emergency nurses
Brazil/2019^7^	Forensic Science International	Cross-sectional	Emergency professionals of the Sergipe Emergency Hospital
United States/2020^35^	Journal of Forensic Nursing	Cross-sectional	Emergency Department health professionals
Turkey/2020^36^	Signa Vitae	Cross-sectional	Emergency nurses
Brazil/ 2020^37^	Society of Trauma Nurses	Cross-sectional	Nurses from the Mobile Emergency Care Service

Figure 4 details the objectives of the studies, as well as the main results on the preservation of traces addressed by health professionals in emergency services.

**Figure 4:** Objectives and syntheses of the main results found in the studies. Pesqueira, PE, Brazil, 2021

Objective	Main results
To address the role of Nursing within an emergency department and the final impact on forensic cases with the evidence preserved^14^.	Emergency professionals are responsible for the evaluation, collection, documentation, and for properly following with the chain of custody.
To present how evidence is collected in the emergency department in a situation of sexual assault^15^.	It is necessary to evaluate, know the rudiments of collection, storage of physical evidence, document injuries by photographic means, avoid biological degradation and follow the chain of custody.
To address obstacles faced for the implementation of the sexual assault response team^16^.	The obstacles were funding, team composition and adequate training of professionals.
To describe the functions of the forensic nurse in the emergency department^17^.	Recognize a potential forensic situation, proceed with collection, preservation, complete documentation of objective and subjective signs, follow up in the chain of custody, notify closest relatives, see the link between patient, physician and investigator.
To review the literature related to the recognition and collection of forensic materials in emergency departments by nurses^18^.	Recognition and collection of evidence should be performed by nurses to forward to the authorities. These professionals must identify physical and non-physical traces, document and maintain the chain of custody.
To evaluate the clinical Nursing practice as a sexual assault examiner in an urgency care center for sexual attacks^19^.	The practice consisted of physical evaluation, gathering evidence and documentation of assault and injuries, screening for Sexually Transmitted Infections and pregnancy, providing treatment and medication, and testifying in court.
To evaluate the Nursing programs as sexual assault examiner^20^.	Medical care was provided 24 hours a day for rape survivors. The minority of the victims received information to understand the medical services, such as pregnancy risk, contraception to prevent pregnancy and the risk of Sexually Transmitted Infections.
To compare the collection of sexual assault nurse examiners (SANEs^*^) in standardized evidence kits with other emergency departments without a SANE^*(^ ^21^.	The examiners trained in the SANE^*^ program perform more complete collections and more compatible with the forensic evidence standards.
To evaluate the education level of clinical practice trauma nurses in a trauma center^22^.	Emergency room professionals received further instructions of forensic protocols on chain of custody maintenance, evidence gathering, and high-quality documentation.
To describe the perception of the importance of forensic role behaviors performed by nurses in emergency departments^23^.	In cases of child physical abuse, the forensic practice must be identified and performed. This is one of the main functions performed by forensic nurses.
To assess the impact of introducing a sexual assault and domestic violence program in the emergency department with forensic evidence collection^24^.	There was a significant increase in pelvic examinations, in the use of the forensic kit, and in the forensic chain. The rates of anogenital lesions, pregnancy and Sexually Transmitted Infections decreased. The flow in the emergency department was optimized.
To develop an evidence-based set of guidelines for forensic evidence collection^25^.	The guidelines address the use of equipment by the professionals; use of the evidence collection kit; general physical evaluation of the patient; adequate collection of fluids, belongings and materials; storage and proper documentation, use of photographs; collection of evidence left by the victim during evaluation.
To describe the types of forensic evidence of firearm injuries and to describe the nurses’ role in treating victims of gunshot wound^26^.	The primary evidence on a gunshot victim is clothing, bullets, gunpowder, and primer. Nursing has the role of acting in the clinic, in communication and collaboration with the authorities.
To describe and compare the forensic knowledge, practice and experiences of nurses and emergency physicians^27^.	Physicians and nurses have the same level of knowledge, and they showed confidence in the collection and documentation of possible evidence.
To compare the care quality indicators in a pediatric emergency department before and after the implementation of a program of pediatric sexual assault nurse examiners^28^.	Implementation of the program maximized care, reduced the time of care, increased the collection of sexual violence kits, and the evaluation and documentation of pregnancy testing and of Sexually Transmitted Infections.
To show the role of collecting and preserving evidence as a Nursing competence of the emergency department^29^.	For collection and preservation, there should be informative evidence (stories, expressions, odors); tangible physical evidence (macroscopic and/or microscopic) through protocols and techniques, using forensic kits; to establish the chain of custody.
To describe the nurses’ views on the forensic care provided to victims of violence and their families in emergency departments^30^.	Most of them knew about the care protocols, cooperated with the authorities and involved families in the victim’s care process.
To identify categories of forensic patients in the intensive care unit and emergency department^31^.	The identification of 27 categories of forensic patients made it possible to properly recognize, document and collect evidence according to each lesion, before offering the necessary care for the patient’s wounds.
To evaluate professionals who worked in 112 emergency centers on how to act in forensic cases^6^.	Most of the professionals were unable to recognize, preserve, protect and did not communicate adequately to the authorities.
To describe the need for a collaborative relationship between the advanced practice of Forensic Nursing in the emergency department and intensive care environments^32^.	Nurses are the link between the patient, the health system and the law. Their function is the recognition, collection, documentation, photodocumentation and completion of reports, so that the evidence is delivered to the authorities.
To address incidents with active snipers in the United States in public places, including hospitals^33^.	Spontaneous reaction, post-event actions and evidence collection procedures should be part of the continuous actions of emergency nurses.
To define Nursing care in cases of trauma on the collection and preservation of forensic evidence in the emergency department^34^.	Nurses should recognize the impact of trauma on the patients, early recognition of evidence, so that collection and preservation is part of the care plan, collaborate with other emergency professionals and guide the patients’ privacy and rights.
To investigate the knowledge and practice of emergency professionals on forensic processes during care for victims of violence^7^.	Half of them were unaware of the collection, documentation and preservation procedures. There was no habit of collecting and documenting objects present in the victim’s body, hospital materials and devices used during the procedures.
To determine the levels of knowledge of emergency department health professionals in the treatment of frequently encountered forensic cases^35^.	There was a low level of forensic training and the professionals, who are not prepared to carry out the practices, must make their own identification record for the authorities.
To determine the Nursing professionals’ knowledge level regarding the approach to cases and forensic tests^36^.	The Nursing professionals did not have the knowledge and technical competence to perform forensic tasks; as they did not receive adequate training for this, they thought that the role would be the Police responsibility.
To evaluate the relationship between nurses’ knowledge and performance of forensic evidence procedures^37^.	Most of the nurses knew the items for documentation and performed them in practice. Only 10.2% knew the procedures for preservation and evidence collection, but less than 1% practiced them.

^*^SANE = Sexual Assault Nurse Examiner

The scope of the scientific production on the preservation of forensic traces by Nursing professionals working in emergency services is presented in five categories: 1) Nursing professionals’ knowledge about the preservation of forensic traces^14^ ^,^ ^16^ ^-^ ^17^ ^,^ ^20^ ^-^ ^22^ ^,^ ^25^ ^-^ ^27^ ^,^ ^29^ ^-^ ^30^ ^,^ ^34^ ^-^ ^35^ ^,^ ^37^; 2) Procedures performed by Nursing to preserve traces in the victim’s body^6^ ^-^ ^7^ ^,^ ^15^ ^,^ ^18^ ^,^ ^20^ ^-^ ^21^ ^,^ ^24^ ^-^ ^26^ ^,^ ^28^ ^-^ ^36^; 3) Procedures performed by Nursing to preserve the victim’s belongings/objects^14^ ^-^ ^15^ ^,^ ^18^ ^,^ ^23^ ^,^ ^25^ ^-^ ^26^ ^,^ ^29^ ^,^ ^32^ ^-^ ^33^ ^,^ ^35^ ^-^ ^36^; 4) Procedures performed by Nursing to document traces^19^ ^,^ ^21^ ^,^ ^24^ ^-^ ^29^ ^,^ ^31^ ^-^ ^37^; and 5) Actions performed by Nursing to maintain the chain of custody^7^ ^,^ ^16^ ^-^ ^17^ ^,^ ^25^. The synthesis of the scientific production is presented in Figure 5.

**Figure 5:** Main recommendations cited for the preservation of forensic traces by health professionals. Pesqueira, PE, Brazil, 2021

*Nursing professionals’ knowledge about the preservation of forensic traces*
● Safety of the professionals
- Use Personal Protective Equipment.
● Firearm
- Remove the projectile from the patient’s body.
● Signs of child abuse
- Identify physical and emotional abuse.
- Collect for investigation purposes.
● Trauma
- Prepare/Follow forensic protocols for collecting and identifying evidence in the emergency room and in the Intensive Care Unit.
● Sexual assault
- Use wound diagrams to collect evidence.
- Collect biological material with a sexual assault kit.
*Procedures performed by Nursing to preserve traces in the victim’s body*
● Firearm
- Wrap the shooter’s hands in a paper bag for gunpowder collection and prime.
- Take photographs of the wounds.
- Do not puncture intravenous access in the hands of the possible aggressor.
- Dry outdoors and store in paper bag the first dressing in gunshot wounds.
● Violence in general
- Collect blood before administration of crystalloids, medications or blood products.
- Store the gastric contents in cans or empty bottles.
- In cases of bites, collect the wound with moistened swabs and photograph the injury site.
- Collect saliva with sterile and moist swab, on the tongue and cheek.
- Collect and store mouthwash water.
- Circle with a marker and take photographs of the wounds or injuries.
● Trauma
- Use povidone iodine to prepare the puncture site.
- Perform cephalocaudal evaluation on the victim.
- Photograph each wound using a scale before and after procedures.
- Collect hair sample.
- Collect dry or wet secretions near or far from the lesions.
- Collect saliva with swabs moistened with sterile water.
● Sexual assault
- Inspect the upper surface of the thighs and photograph injuries.
- Dry outdoors and freeze vaginal, rectum and mouth swab material separately.
- Visually record blood and sperm splashes and hair sample.
- Collect hairs from the head and pubis.
- Protect nails for forensic evaluation.
- If nail scrapes occur, store aseptically.
- Perform pelvic examination in a multidisciplinary approach with a physician.
- Allow shower bath after collection and documentation of injuries.
● Run overs
- Store in paper bags the shards of glass on the victim or on the hospital gurney sheets.
- Collect with adhesives paint, dirt, vegetation, fabric, nails, insects or unknown debris.
*Procedures performed by Nursing to preserve traces in objects*
● Victim’s clothes
- Remove without cuts in the holes of firearm or melee arms.
- Cut along the seams, and store in a paper bag.
- Photograph bloodstains on the clothes.
- Do not allow access and handling of family members and/or friends.
- Allow to dry at room temperature; if not possible, keep the bag open and notify the police.
- Undress the patient with the use of paper sheets in order to find strands of hair and dirt.
● Bed sheets
- Allow to dry at room temperature.
- Store hospital bed sheets in separate paper bags.
● Shoes
- Store each piece of shoe in a separate packaging.
*Procedures performed by Nursing to document traces*
- Record the patient’s condition.
- Perform therapeutic intervention.
- Preserve forensic evidence.
- Report injuries to the competent authorities.
● Firearm
- Observe and document the presence of soot and dust.
- Document/Record the description of the lesions found.
- Document/Record the location of the projectile(s) recovered.
- Document/Record firearm collection.
- Handle gun with gloves and store in paper bag.
- Handle projectiles, preferably with tweezers.
- Store projectile collected from the patient’s body in a plastic cup with gauze.
- Store each projectile in a separate container.
- Identify the container with the patient’s data.
● Sharp-edged weapons
- Handle with the use of gloves.
- Store each weapon in a separate container.
- Store removed needles in empty glass containers.
● Trauma
- Document/Record the patients’ statements accurately.
- Document/Record location, size and appearance of injuries and medical interventions.
- Document/Record the patient’s appearance, behavior, attitudes and concerns.
- Document/Record unusual odors.
● Sexual assault
- The sexual assault collection kit can be frozen for up to 6 months.
*Actions performed by Nursing to maintain the chain of custody*
● Care of the objects collected
- Seal all containers with adhesive tapes, label with the collector’s name, collection date and time.
- Do not discard the projectiles or any item that constitutes evidence.
- Give the guns to the police.
● Care with recording the facts
- Complete the chain of custody form, with all object transfer information.

## Discussion

Preservation of forensic traces is fundamental to solve a case, and Nursing professionals are relevant actors in this process since, in the emergency health services, they are the first to receive victims involved in crime situations. In addition to providing health care, Nursing also has the function of identifying, collecting, storing, documenting and continuing the chain of custody, which contributes both to the effectiveness of the care provided to the victims and to justice^7^. Thus, knowledge and technical capacity in forensic Nursing need to be expanded, and dissemination of the mapping of scientific production in this area contributes to this. 

It is noteworthy, however, that, although general strategies such as insertion of forensic content since the training and qualification of the professionals already trained are relevant, translation of the knowledge must be adapted to regional particularities, given the heterogeneity of the Brazilian context. To this end, there is a need for research studies that investigate the teaching-learning process on the theme in the various Brazilian realities, so that there is support for evidence-based practice in the training of professionals about the forensic aspects.

Most of the studies in this review correspond to the international reality of nurses’ performance in the face of forensic situations, as Forensic Nursing is not a reality in all countries. In Brazil, although Nursing class entities recognize Forensic Nursing, its actual practice in the health services still needs extensive expansion. The national reality found in the sample explains the low implementation of most of the actions related to the preservation of traces, related to the incipient inclusion of this theme, from the training of these professionals to permanent education^7^. In this sense, the procedures adopted in other countries need to be implemented in countries where the forensic scenario is not routine in the Nursing practice, as is the case in Brazil. Thus, the urgent need to include forensic content in the curricula of technical courses, undergraduate courses and specializations in Brazilian Nursing is confirmed. In addition to that, there is a need to expand and strengthen the class struggle so that there is a legal obligation regarding the inclusion of forensic nurses in emergency services, so that they can act in crime cases and in the permanent education of the professionals, as well as collaborate with managerial improvements that favor preservation of traces. 

The lack of training, knowledge and technical competence for forensic tasks by the majority of nurses working in the emergency room was highlighted in the studies. Those who possessed some level of forensic knowledge did not master all stages of the preservation processes, which caused insecurity^18^ ^,^ ^34^ ^-^ ^37^. In Turkey, this scenario was also identified: 80% of the nurses who attended to forensic cases were able to differentiate types of evidence; however, they did not know how to collect, store and refer to the competent authorities^6^. In this context, there is an urgent need for permanent and continuing education aimed at training Emergency Nursing not only to act on topics that are generally addressed, such as the care of trauma victims and clinical emergencies, but that is also consistent with training for the Nursing care to be provided in cases involving crime, with proper preservation of forensic traces. To this end, forensic content should be inserted as mandatory in professional training and updates; it should be covered in selection processes, in residency tests and academic job competitions in the area, so that there is also demand and interest in the professionals for the content, thus becoming agents of the pedagogical process.

Regarding preservation of traces, the most prominent contexts in the studies were about the professionals’ safety, preservation of traces in crimes involving firearms, identification of signs of child abuse, and recognition of traces in cases of trauma and sexual assault^7^ ^,^ ^18^ ^,^ ^20^ ^-^ ^22^ ^,^ ^25^ ^,^ ^27^ ^,^ ^29^ ^,^ ^34^ ^-^ ^35^ ^,^ ^37^. It is inferred that Nursing has sufficient training to prioritize physiological, pharmacological and procedural aspects of such themes, which may even involve subjective issues such as humanization. However, they may be unaware of the judicial consequences of their assistance actions, so that they discard relevant evidence for criminal investigation. This fact was observed in a research study conducted in New Zealand, which investigated nurses in the emergency department and whose results showed limited knowledge about criminal legislation, as well as that 84% reported considering the theme important for their professional practice^38^. These findings point to the need for greater intersectoral and interdisciplinary approximation of Nursing with the transversal legal aspects of emergency health care. Such approximation is presented as the duty and responsibility of Nursing professors and professionals who, in the Brazilian context, recommend/determine the profile of the training for the profession in the National Curricular Guidelines.

In addition to allowing understanding the processes in forensic cases, education for emergency nurses on how to preserve traces enables improvements in patient care. However, training on this theme for Nursing is scarce, including the gap of intersectoral articulation with public safety^7^ ^,^ ^18^ ^,^ ^34^ ^-^ ^36^. In the United States, it is common to offer online training courses, which have already shown a 25% increase in the participants’ knowledge^39^. Therefore, new studies addressing the elaboration, validation, application and comparison of teaching strategies on the theme are relevant to guide the decision-making processes of the professionals involved in Nursing professionals’ education and training.

As for the procedures to preserve traces in the victim’s body, the studies included in this review presented diverse information on this procedure in the most varied crime scenarios, such as firearm violence, trauma, sexual assault and run over cases. The findings corroborate with a literature review that points out the importance of the diverse information contained in the traces of the victim’s and the aggressor’s bodies, which must be collected during the physical examination and need to be carefully stored and documented, for clarification in the criminal investigation^1^. The implementation of these practices implies effective preservation, rich in materials and with lower risk of contamination, and is relevant because the body consists of an area exposed to constant alteration, both by the physiological and metabolic dynamics, and by the actions inherent to the self-care routine, such as bathing and personal hygiene.

For comprehensive care to victims of sexual assault, a detailed physical examination is essential. The Sexual Assault Nurse Examiners (SANE) program, present in some emergency health services in the United States, has nurses specialized in the care of sexual assault victims, and establishes the use of protocol and forensic evidence kits, necessary for the collection of traces^15^ ^,^ ^19^ ^,^ ^21^ ^,^ ^24^ ^,^ ^28^. The program also works with preventive measures for the victims, which include pregnancy tests, emergency gestational contraception and administration of prophylactic drugs for Sexually Transmitted Infections (STIs), as well as emergency contraceptives, clinical and psychological professional monitoring for 72 hours, in addition to storage of the evidence kit for up to six months, until the victim decides to use its content^40^ ^-^ ^41^. It is noted that it is appropriate for Brazilian Nursing and the Federal Nursing Council to implement a similar initiative, in conjunction with the Criminal Investigation Police Department, in order to optimize care and corroborate the holistic view of Nursing care and the equity and resoluteness recommended by the Unified Health System (*Sistema Único de Saúde*, SUS).

In the context of preserving the victim’s belongings/objects, the importance of the clothes as one of the main sources of traces is highlighted, as they may contain physical and biological aspects and components that help elucidate the crime. However, clothing is usually discarded or mischaracterized in emergency care. A study carried out in the United States highlighted the importance of clothing in victims of perforations by firearms and the wealth of information it can present about the crime suspect, the victim, the weapon used and the dynamics adopted at the scene since, for example, gunpowder can be deposited in the tissue and the pattern of blood spatter and holes can determine the projectile’s entry and exit pattern, which may even allow inferring the position of those involved at the time of firing^42^. Thus, the Nursing professional needs to recognize the types of traces in the patient, as well as to know how to proceed with the belongings and clothes that arrive at the emergency room, as they contain relevant information about what happened and need to be preserved.

The studies of the sample pointed to the preservation of traces performed by emergency nurses, which occurred in shoes, bed sheets and other objects of the victim^17^ ^-^ ^18^ ^,^ ^23^ ^,^ ^25^ ^,^ ^29^ ^,^ ^33^ ^,^ ^35^ ^-^ ^36^. These findings differ from those found in a study conducted in Brazil, whose results showed that, although the Nursing professionals recognized the need to preserve traces, it did not occur in such items, due to lack of routine and absence of documentation/registration about the victim’s objects and belongings^7^ ^,^ ^23^. Thus, it is perceived that to strengthen the practice in the Brazilian territory, it is necessary to devise and implement institutional protocols, in order to better guide the forensic practice by nurses working in emergency services.

After the collection stage, documentation of the traces must be done in a thorough and attentive manner by Nursing because it is through this procedure that it will be possible to structure diverse information and prepare arguments to be analyzed in order to solve the crime. In this review, contents were identified that include actions ranging from the record about the patient’s condition to the detailing of the record about the objects found^19^ ^,^ ^21^ ^,^ ^24^ ^-^ ^25^ ^,^ ^27^ ^,^ ^29^ ^,^ ^32^ ^-^ ^35^. Such actions are in line with a study carried out in Portugal, which highlights the relevance of detailing the documentation and record, which must be descriptive and accompanied by a photographic record^43^. Despite being often associated with the bureaucratic and tiring routine, documentation/recording stage, with a wealth of details not only contributes to justice occurring through the resolution of a crime, but also culminates in legal support for the professional Nursing practice and can be triangulated with the professional’s report/testimony, if summoned to give testimony to the Police and/or judicial authorities.

In the chain of custody, follow-up of the stages causes concern to nurses, as it does not consist only in storing the evidence in sealed and labeled containers, but also in the delivery of weapons and projectiles to the authors of the law and in the recording, by stamp and signature, of all the information therein contained^7^ ^,^ ^17^ ^,^ ^25^. A case report for the implementation of the SANE course in Brazil pointed out that the nurses’ actions can contribute to the suitability of the chain of custody in the health services^44^. In the case of Saudi Arabia, nurses’ concern with legal responsibilities, in the face of forensic cases, proved to be a barrier to maintaining the chain of custody^45^. Thus, it is noteworthy that clarification about the stages that make up the chain of custody, as well as the importance of the nurses’ role for the success of such a chain is relevant for the professionals’ awareness and sensitization about the need and importance of their correct performance.

The findings of this study may contribute to the multiplication of information on a topic little explored in the Brazilian reality, provide the argument about the need for intersectoral and interdisciplinary articulation between health and safety, and favor the conduction of new research studies in the area, with a view to promoting the development of forensic protocols in health institutions and implementing training of Nursing professionals in emergency services

The limitations of this scoping review are the methodological heterogeneity of the studies found, which restricted the possibility of comparing the results, and the incipience of studies on the preservation of forensic traces carried out by Nursing, which acts specifically in the pre-hospital emergency context.

## Conclusion

This scoping review made it possible to map diverse evidence on the preservation of forensic traces by Nursing professionals in emergency services. The Brazilian evidence on the topic is limited. Mainly in the international scenario, the studies surveyed pointed out limited knowledge of the Nursing professionals about the theme, whether in the procedures performed by Nursing to preserve traces in the victim’s body, belongings and objects, in the documentation of traces and/or in the actions carried out by Nursing to maintain the chain of custody, especially in situations of aggression, injuries involving firearms, sexual violence, child abuse, and in the assistance provided to trauma victims.
